# Genetic diversity of West Nile virus lineage 2a circulating in *Culex pipiens* vector populations from Bucharest city during the 2024 and 2025 transmission seasons

**DOI:** 10.1186/s13071-026-07561-4

**Published:** 2026-07-18

**Authors:** Sorin Dinu, Florian-Liviu Prioteasa, Aurelian Pintiliescu, Oana-Claudia Albu, Laura-Ioana Popa, Ramona-Ionela Iordache, Maria Condei, Mihaela Oprea

**Affiliations:** 1 Molecular Epidemiology for Communicable Diseases Laboratory, Cantacuzino National Military Medical Institute for Research and Development, 103 Splaiul Independenței, 050096 Bucharest, Romania; 2 Medical Entomology Laboratory, Cantacuzino National Military Medical Institute for Research and Development, 103 Splaiul Independenței, 050096 Bucharest, Romania; 3 Compania Municipală Eco Igienizare București S.A., 2-6 Bacău Street, 052299 Bucharest, Romania; 4 Genomics, Transcriptomics, and Bioinformatics Platform, Cantacuzino National Military Medical Institute for Research and Development, 103 Splaiul Independenței, 050096 Bucharest, Romania

**Keywords:** West Nile virus, Lineage 2a, Cluster B, Genetic diversity, *Culex pipiens* mosquitoes, Bucharest city, Romania

## Abstract

**Background:**

West Nile virus (WNV) is an emerging pathogen in Europe with an unprecedented northward expansion. In Romania, the virus has been endemic for at least three decades, and human infection cases are registered yearly, sometimes culminating in outbreak events. Bucharest, the capital city of Romania, is the largest urban area of the country, encompasses a multitude of mosquito and bird habitats, and has been continuously reported as an active hotspot for WNV transmission. The circulation of lineage 2 has been documented in Bucharest since 2010 in mosquito and human populations. Our study aimed to provide up-to-date information on WNV isolates circulating among mosquito vectors collected in Bucharest.

**Methods:**

Mosquito collection was performed during the 2024 and 2025 WNV transmission seasons at ten sampling sites. WNV was detected in mosquito pools by real-time PCR, and partial and complete genome sequences were obtained and used for inferring phylogeny.

**Results:**

In both study years, WNV isolates belonging to cluster B of lineage 2a were detected in *Culex pipiens* s.l. mosquito pools. Phylogenetic analysis indicated the presence of multiple isolate clusters containing sequences previously obtained from samples collected in Bucharest or elsewhere in Romania, as well as from other European countries. The high genetic diversity of WNV lineage 2a in the studied area is sustained by variations observed between sequences obtained from different sites, between sequences from the same site but in different years, and even between sequences from the same site in the same year.

**Conclusions:**

Close genetic relatedness between the WNV isolates depicted in this study and viruses from European areas with intense WNV circulation in recent seasons, such as Greece, Hungary, Italy, and Russia, or from regions where the virus was recently identified, such as Poland, was demonstrated. A clear trend toward cluster replacement between the two study years was shown.

**Graphical Abstract:**

**Figure:**
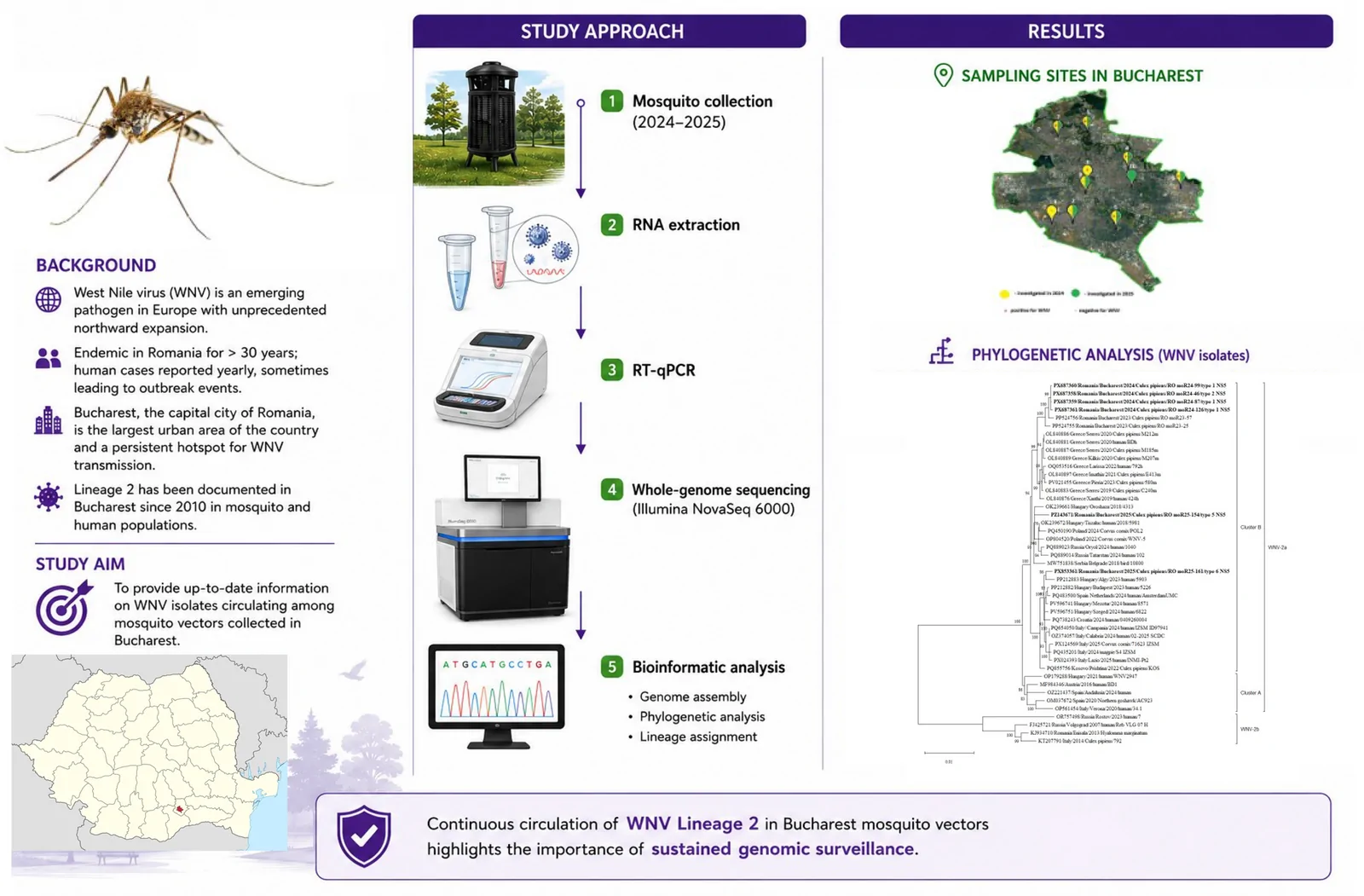

## Background

West Nile virus (WNV), currently classified as *Orthoflavivirus nilense* (family *Flaviviridae*), is an arbovirus maintained in transmission cycles among birds as amplifying hosts, especially species belonging to Passeridae and Corvidae families, and mosquito vectors that belong mainly to the *Culex* genus. WNV is neurotropic and can cause mortality in both birds (corvids being the most susceptible of dying from the disease) and mammals. Although humans represent dead-end hosts for WNV, with only 1% of the infection cases developing severe neuroinvasive disease, the virus represents a significant public health threat in many regions of the world. Nine lineages (1–9) have been described for WNV. Lineages 1 and 2 are known to cause disease in humans with lineages 1a and 2a being responsible for most human cases worldwide [1–4]. Lineage 2a is the most widespread in Europe [4], and it emerged in 2004 in Hungary [5].

Barely known before the 1990s in the Northern Hemisphere, WNV has caused numerous outbreaks in this part of the world in the recent decades. In 1985, Ukraine experienced an outbreak of 38 cases [6]. Romania followed in 1996, with 352 neuroinvasive disease cases and 17 fatalities out of a total of 393 infections reported [7]. An outbreak of 624 cases was recorded in 1998 in Georgia [8] and preceded an extremely severe epidemic in southern Russia between August and October 1999. Approximately 1000 cases were recorded, 40 of which were fatal [9]. In 2010, large epidemics occurred in Europe, with countries such as Greece, Romania, and Russia reporting circulation of different WNV lineage 2 strains [10–14]. The number of WNV infections peaked in Europe, including Romania, during the 2018 outbreak, when the number of cases reported on the continent (2083) exceeded the total number from the previous seven years [15]. WNV subsequently dispersed northward into Europe in countries such as Germany [16], the Netherlands [17], and Belgium [18], with cases of locally acquired infection being recorded in 2025 in Paris, France [19]. The number of human cases reported for the 2025 transmission season in Europe (1112), although smaller than the number recorded for the previous year (1436), was above the average for the past decade (758) [20].

Bucharest city, together with its surrounding Ilfov county, represents Romania’s largest and most densely populated urban region (2.26 million people over an area of 1804 km^2^). This region lies in the Romanian plain and encompasses a multitude of mosquito and bird habitats, such as residential, industrial, and agricultural areas; parks; forests; wetlands; bodies of water; protected areas, including an urban nature park; and unused lands becoming ecological corridors. Since the 1996 outbreak caused by lineage 1 [7, 21], this metropolitan area has been continuously documented as a hotspot for WNV transmission. The evidence for WNV circulation in the Bucharest region and in other parts of Romania was obtained over the years by human, mosquito, bird, and horse studies, with continuous detection of lineage 2 recorded since the 2010 outbreak. Human infection cases are registered yearly in Romania, sometimes culminating in outbreak events [13, 22–30].

We conducted a two-year surveillance study (2024–2025) to detect and characterise WNV isolates circulating in mosquito populations from Bucharest city.

## Methods

### Entomological investigation

Mosquito collections were performed during the 2024 and 2025 WNV transmission seasons (June–September). Ten sampling sites located in Bucharest city, Romania, were investigated (Table 1, Fig. 1). Centers for Disease Control and Prevention (CDC) gravid traps (John W. Hock Company, Gainesville, FL, USA) were used to collect adult mosquitoes. The use of these traps presents several advantages such as: continuous collection both during the day and at night, thus targeting both diurnal (e.g., *Aedes albopictus*) and nocturnal mosquito species; long operating autonomy (for up to one week); very effective in urban settings; they mainly attract pregnant females that have had at least one blood meal, thus increasing the chances of finding WNV positive samples. The specimens were morphologically identified [31], and female mosquitoes belonging to *Culex pipiens* s.l. and *Aedes albopictus* species were pooled into samples of up to 50 individuals according to the date and place of collection. Owing to some limitations, not all the sites were analysed in both years. The samples were stored at − 70 °C until processing for molecular analysis.

**Table 1:** Mosquito collection sites investigated in Bucharest city, Romania, 2024–2025, and the results of West Nile virus testing by real-time PCR

Mosquito collection site	Latitude (N)	Longitude (E)	2024			22025		
			Collection period	*Culex pipiens* s.l.*	Aedes albopictus*	Collection period	*Culex pipiens* s.l.*	Aedes albopictus*
1	44° 29′ 14.40″	26° 4′ 35.40″	21 June–23 September	660/22/1	272/8/0	10 June–10 September	767/23/4	90/2/0
2	44° 23′ 33.6″	26° 4′ 4.43″	21 June–20 September	972/30/0	0/0/0	13 June–26 August	639/21/0	114/3/0
3	44° 25′ 15.82″	26° 4′ 54.62″	28 June–07 August	507/17/0	0/0/0	18 June–15 September	310/10/0	0/0/0
4	44° 26′ 42.25″	26° 8′ 4.21″	21 June–11 September	414/15/2	109/4/0	16 June–10 September	640/22/1	0/0/0
5	44° 23′ 13.96″	26° 7′ 9.19″	18 June–23 September	853/28/8	441/13/0	10 June–10 September	1054/31/3	30/1/0
6	44° 25′ 35.17″	26° 11′ 57.00″″	02 July–11 September	209/7/1	68/2/0	16 June–05 September	129/6/0	0/0/0
7	44° 28′ 54.78″	26° 2′ 32.52″	28 June–10 September	1572/47/8	75/2/0	10 June–05 September	1490/44/4	0/0/0
8	44° 25′ 55.87″	26° 5′ 1.03″	05 July–06 August	342/9/1	0/0/0	NA	NA	NA
9	44° 23′ 31.18″	26° 2′ 36.41″	26–29 June	150/5/0	0/0/0	NA	NA	NA
10	44° 25′ 37.94″	26° 8′ 18.61″	NA	NA	NA	11–15 July	291/9/0	0/0/0
Total				5679/180/21	965/29/0		5320/166/12	234/6/0
				6644/209/21			5554/172/12	

*Number of mosquitoes/number of mosquito pools/number of mosquito pools positive for WNV

**Fig. 1:**
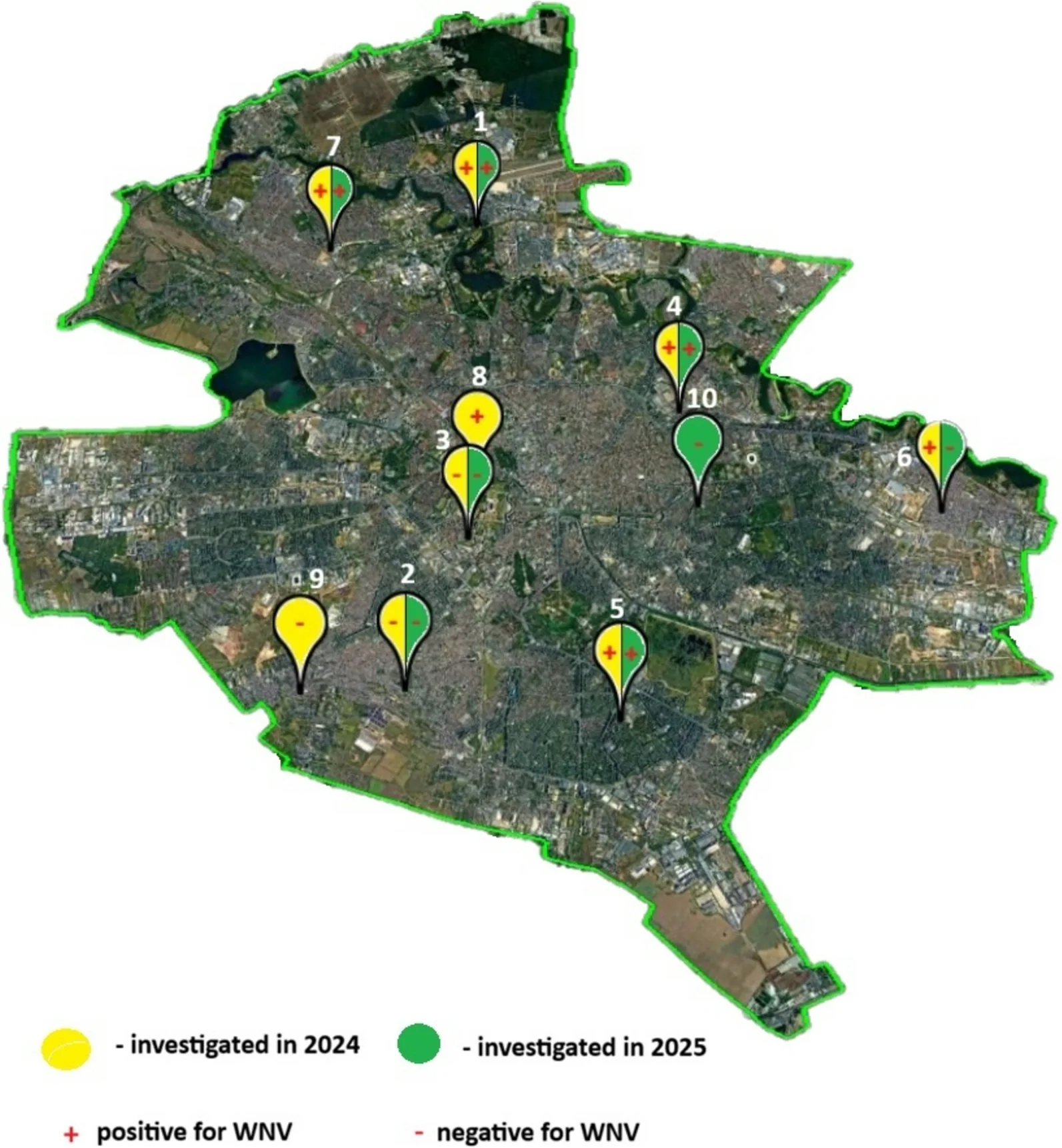
Mosquito sampling sites, Bucharest city, Romania, 2024–2025

### Molecular analysis

Mosquito pools were homogenized in 1 mL sterile PBS 1X, pH 7.4, containing 20% foetal bovine serum, 400 U/mL penicillin, 400 µg/mL streptomycin, and 1 µg/mL amphotericin B, using two glass beads and a Sartorius Mikro-Dismembrator U (2000 rpm, 5 min). The homogenate was centrifuged (14,000 rpm, 10 min, 4 °C) and 140 µL of supernatant were used for RNA extraction with QIAamp Viral RNA Mini Kit (Qiagen, Hilden, Germany). The RNA was eluted in 50 µL AVE buffer provided in the kit.

The WNV genome was detected in the RNA extracts by real-time PCR using a West Nile Virus Real-TM Kit (Sacace Biotechnologies, Como, Italy) and a CFX96 Touch Real-Time PCR Detection System (Bio-Rad, Hercules, CA, USA).

The primers FU2, cFD3 [32], and 9368f [5] were used in a heminested PCR to amplify a fragment spanning the NS5 region of the viral genome. Amplicons were sequenced with a BigDye™ Terminator v3.1 Cycle Sequencing Kit on a SeqStudio Genetic Analyzer (Applied Biosystems, Waltham, MA, USA). Electropherograms were visually checked with BioEdit version 7.7.1 [33].

Double-stranded complementary DNA obtained from total mosquito RNA with SuperScript™ IV First-Strand Synthesis System and Second Strand cDNA Synthesis kits (Invitrogen, Applied Biosystems, Waltham, MA, USA) was used as a template for WNV whole genome sequencing. Libraries were obtained with a Nextera XT DNA Library Preparation Kit and sequenced on a NovaSeq 6000 platform, with 2 × 150 bp reads (Illumina, San Diego, CA, USA). Genome assemblies were generated after the Illumina short reads were mapped to a reference sequence (GenBank accession number HQ537483) with Bowtie2. The consensus (-q 20, -t 0.5, -m 10) was called with the ivar tool (https://usegalaxy.eu/) [34]. Only samples with Ct < 20 generated sufficient reads to call accurate consensus sequences.

Partial NS5 or complete genome sequences were analysed by NCBI BLAST (https://blast.ncbi.nlm.nih.gov/Blast.cgi) and with Genome Detective version 1.4 (https://www.genomedetective.com/app/typingtool/wnv/) [35, 36].

All the NS5 sequences were aligned with BioEdit version 7.7.1 [33] using ClustalW Multiple Alignment option. After aligning, the sequences were trimmed at same length (positions 9451–10012 in Nea-Santa-Greece-2010 genome, GenBank accession number HQ537483) and the nucleotide differences were analysed.

Complete genome sequences were used for inferring phylogenetic relatedness with Mega 12.1.2 software, using the Maximum Likelihood method and Tamura-Nei model, with 1000 bootstrap replicates [37].

Partial and complete genome sequences were deposited in GenBank under the following accession numbers: PX687344-PX687361, PX853355-PX853358, PX853360, PX853361, and PZ143671.

## Results

Between 2024 and 2025, a total of 24,065 mosquito specimens (males and females) were collected. *Culex pipiens* s.l. and *Aedes albopictus* strongly predominated the collections (Table 2). Only the females belonging to the *Culex pipiens* species and some of the females belonging to *Aedes albopictus* species were sorted into pools. Thus, 381 female pools containing 12,198 specimens were analysed for the presence of WNV (Table 1).

**Table 2:** Summary of the mosquito samples collected in Bucharest city, Romania, 2024–2025

Mosquito species	2024		2025	
	Number of mosquitoes	Proportion of species	Number of mosquitoes	Proportion of species
*Culex pipiens* Linnaeus, 1758	8423	62.67	6760	63.62
*Aedes albopictus* (Skuse, 1895)	4996	37.17	3837	36.11
*Aedes vexans* (Meigen, 1830)	10	0.07	22	0.21
*Culiseta longiareolata* (Macquart, 1838*)*	4	0.03	0	0
*Culiseta annulata* (Schrank, 1776)	2	0.015	0	0
*Uranotaenia unguiculata* Edwards, 1913	2	0.015	3	0.03
*Coquillettidia richiardii* (Ficalbi, 1889)	1	0.01	0	0
*Culex martinii* Medschid, 1930	1	0.01	0	0
*Aedes geniculatus* (Olivier, 1791)	1	0.01	3	0.03
Total	13,440	100	10,625	100

The virus was detected by real-time PCR in 33 *Culex pipiens* s.l. mosquito pools (Table 1). WNV was first detected at the beginning of July in both study years. The municipal authorities and the Romanian National Institute of Public Health were promptly informed regarding the risk areas before the first human infection cases occurred. The minimum infection rate, – MIR (number of WNV-positive pools/total specimens tested) × 1000, for *Culex pipiens* s.l. was 3.16 in 2024 and 2.16 in 2025.

A total of 25 samples yielded WNV sequences (Ct values between 14 and 29). Among these samples, six generated complete genome sequences (Ct values < 20). Genome Detective and BLAST analysis revealed that all the viruses detected belonged to cluster B of lineage 2a. When the NS5 fragments obtained for all 25 samples were analysed, six sequence types, hereafter designated 1–6, were identified (Table 3). All the nucleotide differences detected in the studied sequences represented synonymous substitutions (Table 4). BLAST analysis indicated that sequence types 1–4 display high nucleotide identity (99.82–100%) with the NS5 sequences of WNV complete genomes obtained from *Culex pipiens* s.l. mosquitoes collected in 2023 in Bucharest city. Four WNV complete genomes originating from samples collected at three sites investigated in 2024 displayed a type 1 or type 2 NS5 sequence. Due to technical limitations, no genomes displaying sequence types 3 and 4 were obtained. Phylogenetic analysis placed these four genome sequences together with viral genomes identified in mosquitoes collected in 2023 in Bucharest, thus confirming the NS5-based analysis. The cluster also contains sequences from human cases and mosquito samples collected between 2019 and 2023 in Greece. Sequence type 5 was identical to sequences from Hungary (2018), Greece (2018), Poland (2022 and 2024), and Russia (2024) but also with a sequence obtained from a human case in central Romania (2015). A single genome displaying a type 5 NS5 sequence was obtained from a sample collected in 2025. The phylogenetic analysis conducted on this genome sequence confirmed the NS5-based analysis. Sequence type 6 was identical to sequences from Hungary, Italy, Croatia, and Serbia obtained from samples collected between 2023 and 2025, and it displayed high nucleotide identity (99.82%) with sequences from eastern and south-eastern Romania (human and mosquito samples, 2018). One genome harbouring a type 6 NS5 sequence was assembled from a sample collected in 2025 and confirmed the NS5-based analysis (Table 3, Fig. 2). With a single exception, sequence types 1–4 were found only in 2024, whereas sequence types 5 and 6 were detected exclusively in 2025. When the collection sites were considered, none of them displayed the same sequence type(s) in both years. With a single exception, a switch from sequence types 1–4 to sequence types 5–6 was recorded for all the sites investigated. The case of site no. 5 is very interesting, since in both study years, the cocirculation of isolates harbouring two NS5 sequence types was recorded. Additionally, for site no. 7, the cocirculation of two NS5 sequence types was recorded only in 2024 (Table 3).

**Table 3:** Summary of the West Nile virus-positive mosquito samples collected in Bucharest city, Romania, 2024–2025

Mosquito collection site	Sample name	Number of mosquitoes per pool	Collection date	Real-time PCR cycle threshold	GenBank accession number for WNV sequence	WNV lineage/cluster**	NS5 sequence type***
1	24–198	25	17–22 August 2024	20.08	PX687357	2a/B	4
	25–65	36	07–14 July 2025	30.85	NA	NA	NA
	25–133	32	08–13 August 2025	24.25	PX853357	2a/B	5
	25–154	42	26 August–5 September 2025	19.25	PZ143671*	2a/B	5
	25–155	41	26 August–5 September 2025	37.32	NA	NA	NA
4	24–42	30	04–08 July 2024	29.09	PX687345	2a/B	3
	24–56	28	08–11 July 2024	21.86	PX687348	2a/B	3
	25–150	33	26 August-5 September 2025	25.86	PX853358	2a/B	1
5	24–29	42	02–05 July 2024	26.74	PX687344	2a/B	1
	24–62	29	08–11 July 2024	28.78	PX687349	2a/B	2
	24–63	29	08–11 July 2024	24.62	PX687350	2a/B	1
	24–99	32	11–16 July 2024	18.70	PX687360*	2a/B	1
	24–101	31	11–16 July 2024	23.75	PX687352	2a/B	1
	24–107	38	18–22 July 2024	27.22	PX687353	2a/B	1
	24–109	38	18–22 July 2024	32.80	NA	NA	NA
	24–110	38	18–22 July 2024	22.81	PX687354	2a/B	1
	25–121	34	13–18 August 2025	30.91	NA	NA	NA
	25–161	40	26 August–5 September 2025	19.68	PX853361*	2a/B	6
	25–164	40	26 August–5 September 2025	29.63	PX853360	2a/B	5
6	24–106	29	18–22 July 2024	29.56	NA	NA	NA
7	24–46	35	05–09 July 2024	17.35	PX687358*	2a/B	2
	24–47	34	05–09 July 2024	18.65	PX687346	2a/B	2
	24–48	34	05–09 July 2024	24.17	PX687347	2a/B	2
	24–50	34	05–09 July 2024	30.31	NA	NA	NA
	24–87	45	11–16 July 2024	14.36	PX687359*	2a/B	1
	24–88	45	11–16 July 2024	17.94	PX687351	2a/B	2
	24–117	36	22–24 July 2024	19.77	PX687355	2a/B	1
	24–195	35	14–17 August 2024	25.61	PX687356	2a/B	1
	25–106	40	31 July–04 August 2025	23.41	PX853355	2a/B	5
	25–107	40	31 July–04 August 2025	22.89	PX853356	2a/B	5
	25–158	33	26 August–05 September 2025	32.17	NA	NA	NA
	25–159	33	26 August–05 September 2025	35.42	NA	NA	NA
8	24–126	37	26–30 July 2024	15.19	PX687361*	2a/B	1

NA: not applicable; *complete genome sequence; **nomenclature proposed in [4]; *** Six types of NS5 nucleotide sequences were detected in the analysed data set

**Table 4:** Single nucleotide polymorphisms observed in the NS5 sequence types identified in the analysed data set

NS5 sequence type	Nucleotide position*							
	9531	9573	9618	9663	9822	9840	9918	10,008
1	C	G	G	C	T	G	C	A
2	C	G	G	C	C	G	C	A
3	C	G	G	T	C	G	C	A
4	C	G	G	C	C	T	T	A
5	T	G	C	C	C	G	T	G
6	T	A	C	C	C	G	C	A

*According to the Nea-Santa-Greece-2010 genome, GenBank accession number HQ537483

**Fig. 2:**
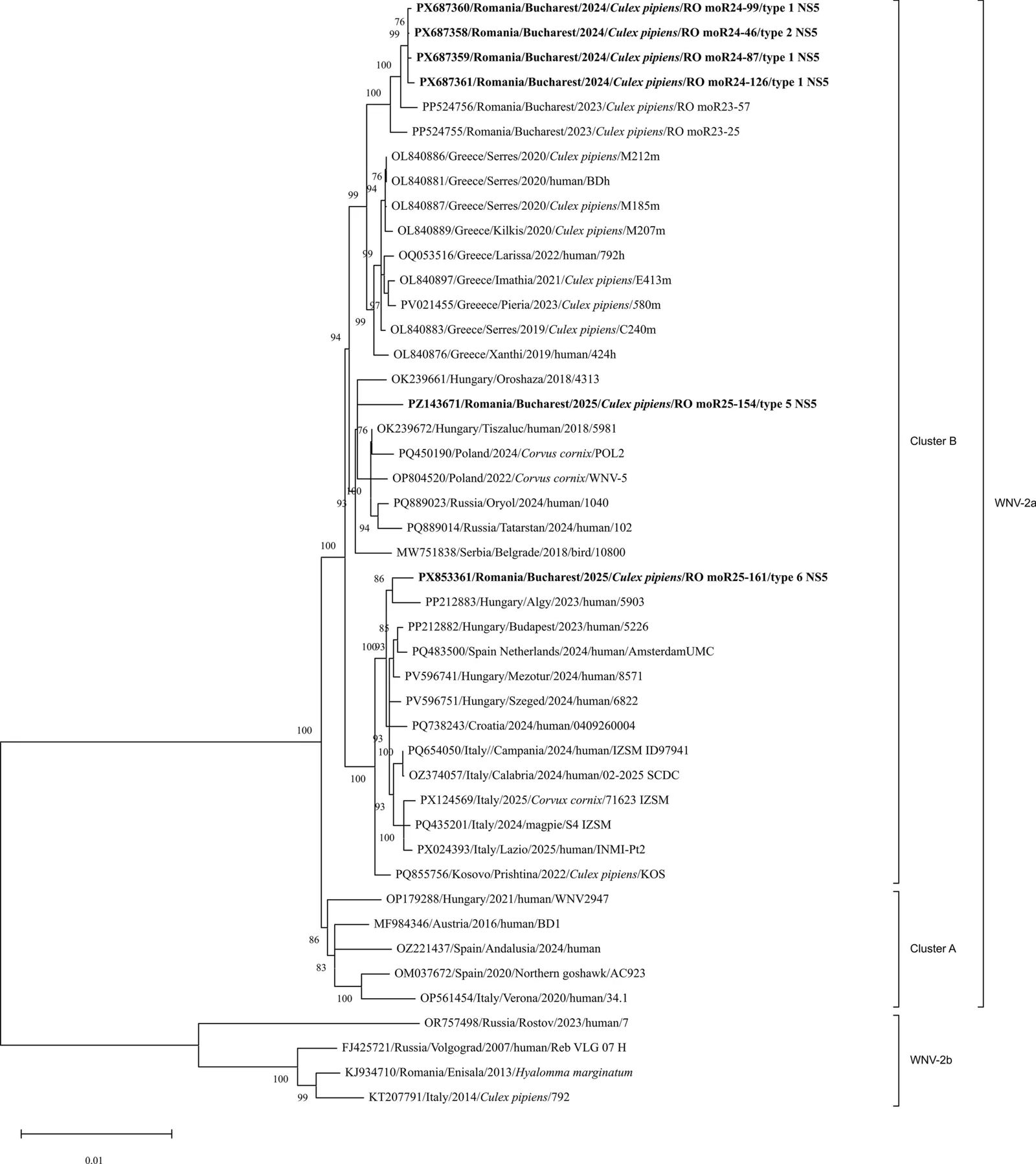
Phylogenetic analysis of West Nile virus isolates detected in *Culex pipiens* s.l. mosquitoes collected in Bucharest city, Romania, 2024–2025. Analysis was conducted on the entire polyprotein coding sequence of the West Nile virus genome (10,305 nt) using the Maximum Likelihood method and the Tamura-Nei model of nucleotide substitutions. The rate model allowed for 70.97% of sites to be evolutionarily invariable (*I*). The percentage of replicate trees in which the associated taxa clustered together in the bootstrap test (1000 replicates) is shown next to the branches (values < 70% are not shown). The names of the sequences obtained in this study and their corresponding NS5 types assigned herein are shown in bold. Nomenclature for lineages and clusters was proposed in [4]

## Discussion

Serological data indicated prolonged WNV activity in southern Romania many years before the unprecedented epidemic occurred in 1996 in this part of the country [8]. Afterwards, the circulation of WNV in the human population was serologically documented almost yearly in Romania. During the 2010 outbreak, the expansion of WNV in the western, central, and eastern regions of Romania was recorded and associated with lineage 2b [13], which further circulated and was detected in mosquito and human populations in this country [24]. Lineage 2a, predominating in Europe [4], began to replace lineage 2b in Romania approximately 2015–2016 [25] and continued to circulate in this country until 2023 [29, 38], causing human infection cases and even outbreaks, the most prominent being the one in 2018 (277 cases) [15].

Our study aimed to provide up-to-date information on WNV isolates circulating in mosquito populations from Bucharest city, a major WNV hotspot in Romania and Europe, during the 2024 and 2025 transmission seasons.

Ten mosquito collection sites located mostly in residential areas were investigated. WNV was detected only in *Culex pipiens* mosquitoes, although our previous study found the virus in *Aedes albopictus* specimens collected in 2017 at two collection sites, which were also investigated in the present work [29]. MIR values calculated for the *Culex pipiens* s.l. collected in 2024 and 2025 in the study area were comparable with those reported for the 2021–2023 period [29], but with an increase for 2024. Eighteen human cases of WNV infection were recorded in both study years in Bucharest city, and a maximum of twenty cases were recorded each transmission season between 2021 and 2023 [39–43]. Blood meal analysis conducted on *Culex pipiens* mosquito populations in the Bucharest metropolitan area showed that the two biotypes of this species shape differently the urban transmission cycle of WNV: the *pipiens* biotype acts as an enzootic/epizootic vector because of its bird feeding preference, whereas the *molestus* biotype is a bridge vector [44].

Genotyping showed that WNV isolates identified in this study belong to cluster B of lineage 2a. However, variations observed between NS5 sequences obtained from different sites, between sequences obtained from the same site but in different years, or even between sequences obtained from the same site in the same year indicate complex local evolution of WNV marked by multiple strain introductions and replacement events. Importantly, a change in the circulating isolates between the two years analysed was observed based on partial sequence analysis. Lineage 2a isolates circulating in Europe are monophyletic and are the result of a single introduction event occurring in Hungary approximately 2002–2004. After their introduction in Europe, lineage 2a isolates diverged into two clusters that spread divergently on the continent. Cluster A emerged around 2006 in Austria and spread to the northwest and western regions, whereas cluster B emerged around 2007 in Hungary and dispersed to south-eastern countries [4]. In Romania, lineage 2a isolates belonging to cluster B were first reported in 2015–2016 in human patients from central Romania and Bucharest city but also in mosquito samples collected in the Danube Delta and Bucharest city during the same period [25]. Cluster B isolates were continuously detected in Romania until 2023 in human, mosquito, and bird samples. The only two detections of cluster A isolates in Romania where in 2020, in *Culex pipiens* s.l. mosquito populations from Bucharest city [29].

The WNV genome sequences (GenBank accession numbers PX687358-PX687361) obtained from samples collected in 2024 during this study were similar to the genome sequences obtained in 2023 from mosquito samples collected in Bucharest city. This cluster also contained isolates obtained from human and mosquito samples collected in Greece between 2019 and 2023. Greece represents one of the most active WNV hotspots in Europe and in both 2024 and 2025 it ranked as the second-most affected country on the continent [20, 42]. Greece contributed the highest number of lineage 2a sequences in Europe, with all belonging to cluster B. It was also shown that cluster B tends to jump more frequently between countries than does cluster A. Approximately 19 transmission events between Greece and neighbouring countries occurred in the past decade, with Romania being among the countries with the most frequent transmissions to Greece (*n* = 4), after Hungary and Serbia; however, the lineage 2a transmission networks in Europe are intricate, jumps between Romania and Greece occurring bidirectionally [4].

On the other hand, the genomes obtained from the samples collected in 2025 grouped in two other clusters, indicating changes in the WNV strains circulating in Bucharest city during the last transmission season and thus confirming the partial NS5 sequence analysis. One genome (GenBank accession number PZ143671) obtained from samples collected in 2025 is related to genomes of human isolates circulating in Hungary in 2018, when the most severe outbreak ever recorded by this country took place [45]. However, based on the publicly available NS5 sequences, similar isolates have been circulating in central Romania (human cases, 2015 and 2018), Bucharest city (mosquito sample, 2017), and eastern Romania (mosquito samples, 2018) [25, 29, 38]. More recent sequences related to this Romanian genome are those from Poland (2022 and 2024) and Russia (2024). The sequences from Poland were obtained from hooded crow samples (*Corvus corone cornix*) collected in 2022 and 2024 in the capital city Warsaw [46, 47]. Interestingly, the first probable case of locally acquired WNV infection was reported by Poland in 2024 [42]. In 2024, 38 constituent entities of Russia reported human cases of WNV infection and an increase in the morbidity rate. An expansion of WNV was also observed in Russia, with nine constituent entities officially reporting locally acquired infections for the first time. Lineage 2 WNV was predominantly circulating in the European part of Russia [48].

The second genome sequence (GenBank accession number PX853361) obtained from mosquito samples collected in Bucharest city in 2025 clustered with genome sequences from Hungary, Italy, and Croatia obtained from samples collected during the last transmission seasons. Notably, based on the limited information provided by the NS5 sequence available from previous years, similar isolates have been circulating in Romania since at least 2018. Those Romanian isolates clustered with isolates that originated after a novel introduction of WNV from Hungary to Greece during the 2018 outbreak [29, 49]. This 2025 isolate is closely related to isolates identified in Italy in 2024 (Calabria and Campania Regions) and 2025 (Lazio Region) [50, 51]. Italy recorded the greatest number of WNV human infection cases in Europe both in 2024 and in 2025. In 2025, the country experienced a massive human outbreak with 779 confirmed cases and 72 fatalities, which represents the highest number ever recorded by this country in a year. Most cases (267) were reported from the Lazio region, where human cases had not been previously reported [20, 42, 50]. Similar isolates were also detected in 2024 in Croatia [52], a country that reported a spread of WNV during that transmission season [53].

The continuous circulation and genetic diversity of WNV lineage 2 have been documented for more than a decade in the studied area [13, 24, 25, 29] and confirmed by the data presented here. The complex eco-epidemiology and genetic diversity of WNV in a certain territory are shaped by several factors, such as (i) climatic variables affecting the dynamics of vector populations, speeding up the viral replication rates in mosquitoes, increasing bird-mosquito interactions, and contributing to the endemization of WNV; (ii) agriculture accelerating WNV dispersal velocity; (iii) urbanization favouring higher abundance of *Culex pipiens* populations; (iv) wetlands and protected bird habitat areas displaying high density of avian reservoirs; and (v) new WNV introduction events driven by migratory birds [4, 54–57]. The studied area sums up all these conditions: Bucharest city acts as heat island and increasing in air temperature and reduction of rainfall were recorded; this area represents the largest urban region of the country in terms of population size and surface, but is also surrounded by a county with intensive agricultural activities; it exhibits numerous and diverse bird habitats, including protected areas; and the region is positioned along the Black Sea-Mediterranean migratory bird flyway.

## Conclusions

This mosquito surveillance study, conducted during 2024 and 2025 in Bucharest city, an important hotspot for WNV transmission in Romania and Europe, highlighted the genetic diversity of the circulating isolates, reflected in the existence of multiple sequence clusters. Relatedness with older isolates identified in the same city or in other regions of Romania was shown; however, phylogenetic analysis indicated also close relationships with viruses from areas with intense WNV circulation in the last seasons, such as Greece, Hungary, Italy, and Russia, or from regions where the virus was recently identified, such as Poland. A clear trend toward cluster replacement between the two study years was shown. WNV genomic surveillance based on entomological studies, such as the present study, is useful for detecting the virus before spillover in the human population occurs and for guiding the implementation of targeted prevention and control measures for maintaining human and animal health. However, an integrated One Health genomic surveillance approach that also includes obtaining and analysing viral sequences from humans, bird reservoirs, and other vertebrates is desirable for a better understanding of the WNV transmission cycle in the studied region.

## Data Availability

The datasets generated and analysed are available from the corresponding author on reasonable request. Nucleotide sequences obtained in this study are available in GenBank under the accession numbers: PX687344-PX687361, PX853355-PX853358, PX853360, PX853361, and PZ143671.
